# Supplementary material for: Using xPIRT to Record Pharmacy Interventions: An Observational, Cross-Sectional and Retrospective Study
Source: Healthcare (Basel). 2022 Dec 5;10(12):2450. doi: 10.3390/healthcare10122450 (PMC9778595; doi:10.3390/healthcare10122450)
Supplement: Supplementary file 1 [file healthcare-10-02450-s001.zip › healthcare-2085183-supplementary.docx]

**Supporting Information**

Using xPIRT to record pharmacy interventions: an observational, cross-sectional and retrospective study

Rafael Baptista ^1,^*, Mary Williams ^2^ and Jayne Price ^1^

^1^ Medicines Management, Powys Teaching Health Board, Hafren Ward, Bronllys Hospital, Brecon, LD3 0LU, UK.

^2^ School of Pharmacy & Pharmaceutical Sciences, Cardiff University, King Edward VII Avenue, Cardiff CF10 3NB, UK.

***** Correspondence: rafael.baptista@wales.nhs.uk;

Table S1 – Description of topics on xPIRT.

| Item | Description |
| --- | --- |
| Date | Date when interventions took place. Can be recorded retrospectively. |
| Hospital and Ward | Ward-specific interventions allow targeting a particular group of prescribers. |
| Contributor | Staff that recorded the intervention. Important to continuing professional development and to track staff engagement with tool. |
| Medicines | Data is categorised by class of medicines, which will relate to the incidence or PIs. |
| Type of intervention | Data is organised by type of intervention, which will relate to the incidence or PIs. |
| Timeline | Interventions can be done on admission, during hospitalisation or prior to discharge. |
| Severity | Intervention severity follows the criteria established by the ScHARR (University of Sheffield School of Health and Related Research) cost model. |
| Acceptance | Acceptance rates for pharmacy recommendations by prescribers |
| Yellow Card completed | Drug adverse reaction report completed |
| DATIX completed | Incident report completed |
| Brief description | Description of each intervention in one or two sentences. This will aid understanding the intervention and recommendations offered to the prescribers. |

Supplementary Table 2 – Intervention description for each error severity.

| Categorisation | Description | Cost |
| --- | --- | --- |
| Potentially lethal | The medication serum level would be in a severe toxicity range (eg. serum theophylline concentrations greater than 30 micrograms per mL).  Dose of chemotherapy agent prescribed is more than 10 times the normal dose.  The drug/dose being administered has a high potential to cause cardiopulmonary arrest or a life-threatening reaction.  The dose of a potentially life-saving drug is too low.  The dose of a drug with a very low therapeutic index is too high, 10 times the normal dose (e.g warfarin, levothyroxine, digoxin, lithium carbonate, phenytoin, theophylline, cyclosporin, tacrolimus, heparin, vancomycin, gentamicin, valproic acid, amiodarone, sotalol, phenobarbital, methotrexate, tamoxifen, carbamazepine, teicoplanin).  Allergy box not completed, with patient with a previous history if anaphylaxis | £1085-£2120 |
| Serious | May increase patient length of stay.  The dose of a drug with a low therapeutic index is too high (4 to 10 times the normal dose, or above normal levels but below toxic levels).  The route of drug is inappropriate, with the potential of causing a severe adverse reaction.  The dose of the drug is too low or too high for a patient who is in acute distress*.  Error of omission whereby patient’s usual medication is not prescribed either on admission, during a rewrite or on discharge, causing acute distress*.  Drug monitoring (e.g. blood tests) is requested/suggested for patient in acute distress*.  Drug-drug interaction or drug-disease interaction when patient is in acute distress*.  The name/dose of the drug is misspelled/illegible, leading to the wrong drug/dose to be administered, causing acute distress*.  Prophylaxis for venous thromboembolism is not prescribed or risk assessed for patients that would require it.  Allergy box not completed, consequently causing an inappropriate medication to be prescribed with potential to cause acute distress to patient*. | £713-£1484 |
| Significant | Will not increase patient length of stay.  The dose of the drug with low therapeutic index is too high (half to four times the normal dose).  The dose of the drug is too low or too high for a patient with the condition being treated, but not in acute distress.  Wrong laboratory studies are ordered.  Drug prescribed/administered with wrong route/dose/frequency/form for the condition being treated, not causing acute distress.  Errors of omission whereby patient’s usual medication is not prescribed either on admission, during a rewrite and on discharge, not causing acute distress.  Suggest reduction of costs/deprescribing/formulary choices.  Medication suggested for untreated condition.  Drug-drug interaction or drug-disease interaction when patient is not in acute distress.  Duplicated therapy prescribed with potential for increased adverse effects.  Request/suggestion of laboratory studies for medication monitoring when patient is not in acute distress.  Allergy box not completed with low risk of inappropriate medication being prescribed  Alert for medication review or stop date, when administration has gone beyond the expected course duration but without adverse outcome.  Prophylaxis for venous thromboembolism is not prescribed or risk assessed, but patient does not require this.  Suggests positive organisational impact (e.g. time saving, improved security, facilitating quicker discharges, training nursing staff).  Suggests an alternative form/frequency/times of medication, to improve patient's compliance or therapeutic effectiveness.  The name/dose of the drug is misspelled/illegible creating a risk that the wrong drug might be dispensed, but not causing acute distress. | £65-£150 |
| Minor | Duplicate therapy was prescribed without potential for increased adverse effects.  The wrong route was ordered without potential for toxic reactions or therapeutic failure.  The order/prescription lacked specific drug, dose, dosage strength, frequency, route or frequency information, with low risk for any adverse effect.  Illegible, ambiguous or non-standard abbreviations used but not leading to potential medication error  The name/dose/route of the drug is misspelled/illegible but unlikely to create risk for the patient.  Missing signatures in drug chart.  Alert for a stop/review date where administration is still within the appropriate date. | £0-£6 |

* acute distress: events linked to a specific and significant adverse outcome.

**Supplementary Figure 1 –** Flow chart with process pathway following a pharmacy intervention.


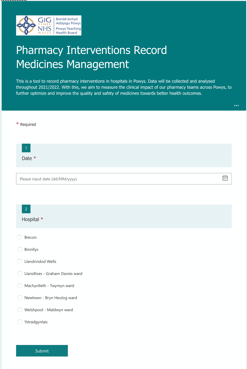

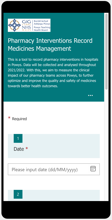

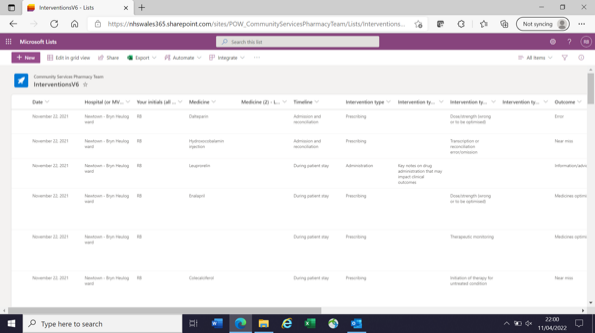

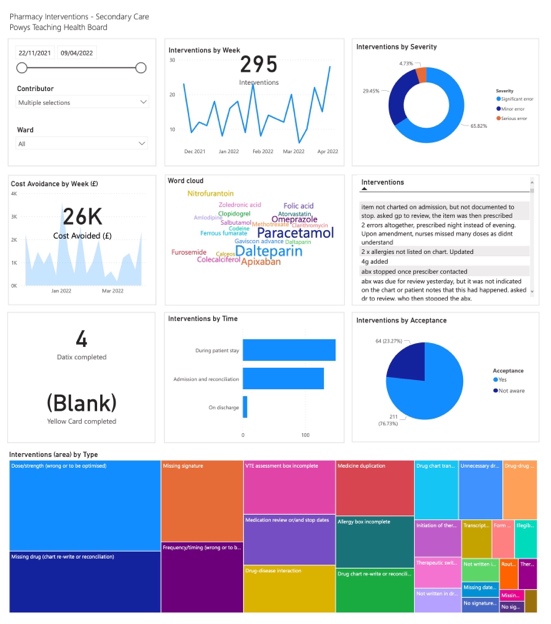


Microsoft Forms

Microsoft Lists.

Data is organised in an automatic table that can be editable anytime.

Microsoft Power BI.

Interactive infographics are available to the team. Data is updated hourly, based on xPIRT List.

xPIRT

xPIRT List

xPIRT Dashboard

xPIRT Toolkit
